# First person – Jun-yi Zhu and Xiaohu Huang

**DOI:** 10.1242/dmm.049329

**Published:** 2021-11-19

## Abstract

First Person is a series of interviews with the first authors of a selection of papers published in Disease Models & Mechanisms, helping early-career researchers promote themselves alongside their papers. Jun-yi Zhu and Xiaohu Huang are first authors on ‘ Pharmacological or genetic inhibition of hypoxia signaling attenuates oncogenic *RAS*-induced cancer phenotypes’, published in DMM. Jun-yi is an assistant professor in the lab of Zhe Han at the University of Maryland School of Medicine, Baltimore, MD, USA, investigating the use of *Drosophila* as a model to study human disease mechanisms and treatment approaches. Xiaohu is a postdoc in the same lab, investigating gene functions in cardiovascular development and genetic diseases.


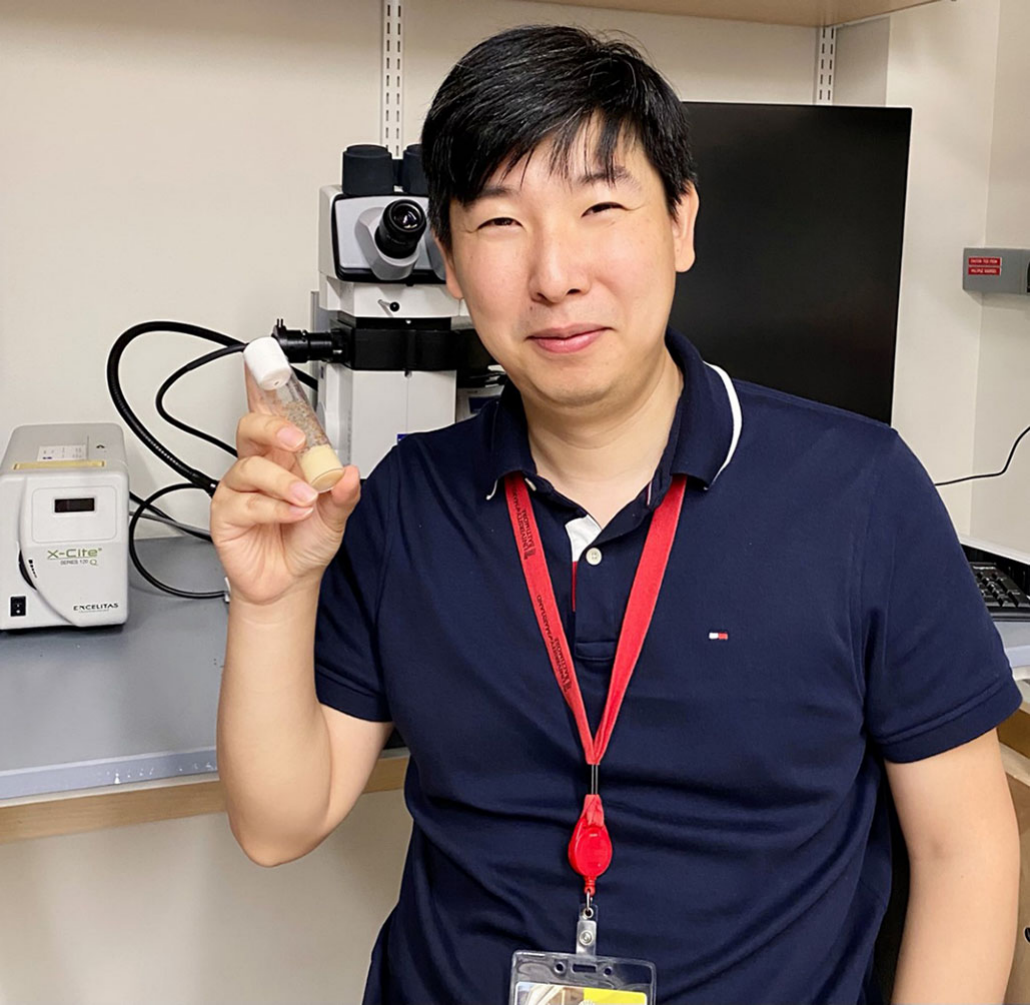


**Jun-yi Zhu**


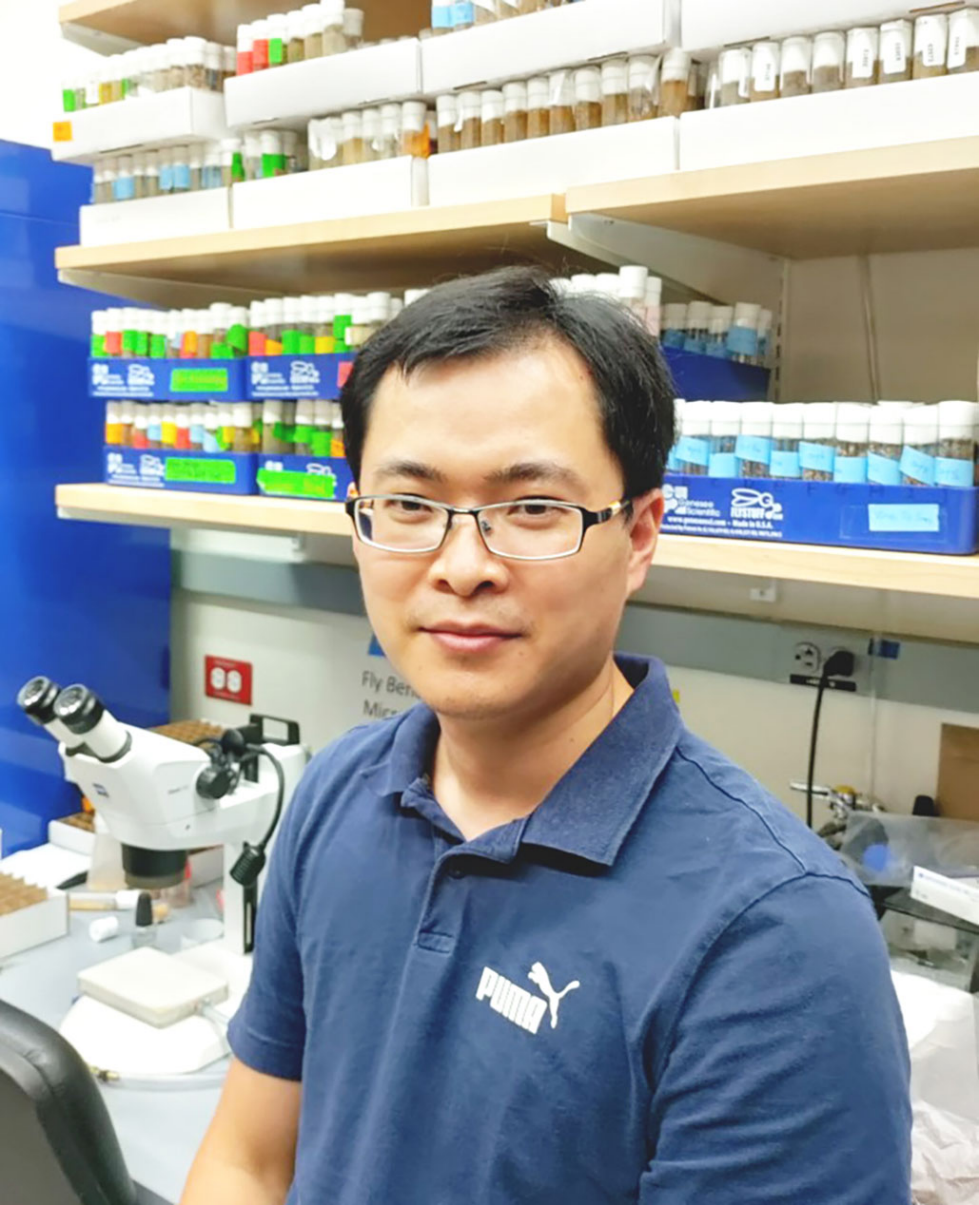


**Xiaohu Huang**

**How would you explain the main findings of your paper to non-scientific family and friends?**

**JZ:** Leukaemia is a type of blood cancer caused by an abnormal rise in the white blood cells in the human body. Currently, lots of research has found that a single gene mutation, like *RAS*, can cause leukaemia. However, there is no effective medication that can be used to treat *RAS*-induced leukaemia. Here, we generated a fly leukaemia model induced by expressing one human *KRAS* mutation. We found that this animal exhibits a dramatic increase in blood cells. Through genetic and drug screens, we identified that the drug echinomycin can cure leukaemia in our fly model. This finding was validated in human leukaemia cell lines and a mouse model. Our study may be of therapeutic benefit for *RAS*-induced leukaemia.

“[…] the hypoxia pathway can be a therapeutic target and echinomycin is a good candidate for the treatment of cancer patients with *KRAS* mutation.”

**XH:** Cancer is the second-leading cause of death following heart disease, both in the United States and around the world. The main reason for this is that there is no effective treatment at all in most cases, which is a result of the lack of understanding of the mechanisms of different cancers. In our study, we first established cancer models in the model organism fruit fly by using mutated human gene *KRAS*, and then we performed both genetic and drug screens to find possible therapeutic targets and drugs for treatment. We found that the hypoxia pathway played a key role in mutant *KRAS*-induced tumours and a small molecule, echinomycin, can effectively inhibit tumour growth, which indicates that the hypoxia pathway can be a therapeutic target and echinomycin is a good candidate for the treatment of cancer patients with *KRAS* mutation.

**Figure:**
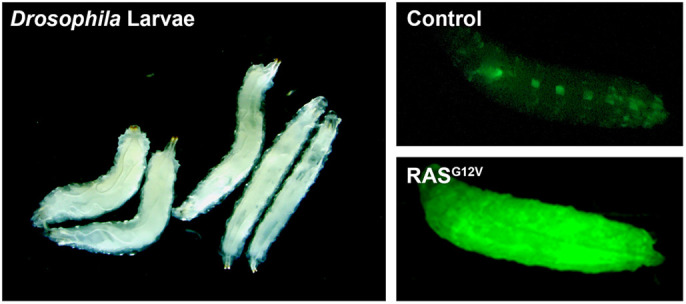
Left: Generation of a *Drosophila* leukaemia model by expressing oncogenic human *KRAS^G12V^*. Right: Haemocytes expressing GFP.

**What are the potential implications of these results for your field of research?**

**XH:** I think many people in both the research and industry fields have a bias for using the fruit fly as a model to establish human diseases and then for a drug test. Our studies in fruit fly demonstrated that a mutated human gene also caused similar disease phenotypes to those found in human patients, and showed that it can be used for drug tests/screens, which has been further validated in a mouse model. Therefore, we hope that this research provides a convincing example of how feasible the fruit fly is as a model organism in modelling human disease and drug tests. Per the advantages of this model, I hope that more and more researchers can choose fruit fly for human disease modelling, drug testing and high-throughput drug screening.

**What are the main advantages and drawbacks of the model system you have used as it relates to the disease you are investigating?**

**JZ:** The fly has been an important model for studying organ development and disease due to remarkable conservation in gene function across species. This, together with its rapid life cycle, high reproductive rate and unparalleled genetic tools, makes the fly an excellent system in which to conduct *in vivo* screens for genes and drugs that suppress different kinds of human disease, not just leukaemia. Though the fly has similar features to humans in blood cells, the main drawback of the fly is the lack of some important types of immune cells, such as T-cells and B-cells, which means that the immune response is different from that of humans. To overcome this disadvantage, the fly work needs to be validated in human cell lines or a mouse model.

**XH:** The main advantages for fruit fly as a model system are its short life cycle, large amount of progeny, low cost and plenty of available genetic resources. It is pretty easy to establish a transgenic or knockdown/knockout fly line in a short time when compared with other model systems. For the drawbacks of the fly system, it cannot be used for the disease modelling of some organs/tissues. For example, only the innate immune system has been revealed in the fly so far, so it cannot be used for the modelling of adaptive immune system-related diseases. In addition, the fly is small, so it will be challenging to collect enough samples for some biochemistry experiments.

**What has surprised you the most while conducting your research?**

**JZ:** Since there is no effective drug to treat leukaemia caused by *RAS* mutation and the *RAS* gene itself is involved in many molecular pathways, we were slightly worried that we could not attenuate the *RAS*-induced leukaemia phenotype through a genetic screen or a drug screen. Therefore, it was an encouraging surprise to find that, in our genetic screen, we identified several different genes that can rescue the *RAS*-induced leukaemia phenotype. In addition, the targets of echinomycin we identified from the drug screen were also found in our genetic screen. It is especially amazing to find the relationship between the two independent screens.

**XH:** When we express a human gene or disrupt the expression of a gene in a fly, the phenotype is usually obvious and easy to catch. In this case, it will help us to easier explore the function of a gene or the mechanism of how a mutated human gene causes disease.

**Describe what you think is the most significant challenge impacting your research at this time and how will this be addressed over the next 10 years?**

**XH:** First, the functions of most human genes are not known. Second, we do not know how the change in a specific gene causes human diseases, especially rare diseases. So, it will be critical to understand the functions of genes in both normal and diseased conditions. The fly model will provide a cost-effective and fast platform for these purposes.

**What changes do you think could improve the professional lives of early-career scientists?**

**XH:** First, I think the department or school should provide some training programs to provide some guidance on how to decide between academia and industry. Second, there should be some platforms/opportunities for early-career researchers to communicate with senior investigators. Third, direct mentor(s) should discuss career plans with their trainee regularly, to help mentor(s) arrange/assign appropriate projects, which will benefit their trainee's future career.

**What's next for you?**

**JZ:** First, in the current paper, through a genetic screen, we found that inhibiting the hypoxia pathway can rescue the leukaemia phenotype in our fly model. However, we still have no idea how this happened. Next, I would like to figure out the molecular mechanisms of how the hypoxia pathway works in *RAS* mutation-induced leukaemia. Second, leukaemia can be caused by a different gene, for example BCR-ABL and AML1-ETO. The disease mechanism and treatment medications for them are different. More fly leukaemia models need to be established and used to provide potential therapeutic benefits for leukaemia.

**XH:** In this study, we established a leukaemia model by using mutant human *KRAS*. In addition to leukaemia, *KRAS* has been a major driver of colorectal cancer, lung cancer and other cancers, so next we will establish tissue-specific tumours by using different *KRAS* mutants. We will also establish more human cancer models in the fly with mutated human genes and explore their functions in cancer initiation and progression.
